# Subarachnoid Hemorrhage With Multifocal Cerebral Aneurysms in a Patient With Crohn’s Disease and Sjögren's Syndrome: A Case Report and Literature Review

**DOI:** 10.7759/cureus.35585

**Published:** 2023-02-28

**Authors:** Mohammad Abu-Abaa, Ghassan Al-Qaysi, Ahmed Hassan, Salman Kananeh

**Affiliations:** 1 Internal Medicine, Capital Health Regional Medical Center, Trenton, USA

**Keywords:** covid-19, aneurysmal subarachnoid hemorrhage, crohn’s disease (cd), ruptured cerebral aneurysm, primary sjogren’s syndrome

## Abstract

Only a few reports of the association between Crohn’s disease (CD) and Sjögren's syndrome (SS) have been documented in the medical literature. Herein, we are presenting a 61-year-old female patient who presented with subarachnoid hemorrhage (SAH). She has a past medical history of primary SS on no active treatment, and CD in remission while on maintenance immunotherapy. She also tested positive for COVID-19. Computed tomography angiography (CTA) brain as well as cerebral angiogram revealed multifocal cerebral aneurysms. Successful coiling was achieved with a cerebral angiogram. This case serves to add to a limited body of reported cases and remind clinicians of the association between SS/CD and cerebral aneurysms. Herein, we review the literature regarding this association and also the effect of immunotherapy and COVID-19 on the progression of cerebral aneurysms.

## Introduction

Non-traumatic subarachnoid hemorrhage (SAH) is most commonly the result of cerebral aneurysm rupture in 85% of cases [1]. The association between cerebral aneurysm with Crohn’s disease (CD) has been previously suggested [2]. Central nervous system (CNS) vasculitis is a rare extraintestinal manifestation of CD [2]. Clinical neurological manifestations in CD include stroke, myelopathy, myopathy, peripheral neuropathy as well as vascular irregularities including stenosis and occlusion [3]. Other manifestations can also include cerebral venous thrombosis and demyelinating disease [4]. On the other hand, Sjögren's syndrome (SS) is an autoimmune disease characterized by periductal lymphocytic infiltration mainly affecting salivary and lacrimal glands [5]. The most common neurological manifestation of SS is peripheral neuropathy [6]. COVID-19 has also been suggested as an inciting factor for SAH [1].

## Case presentation

**Figure 1:**
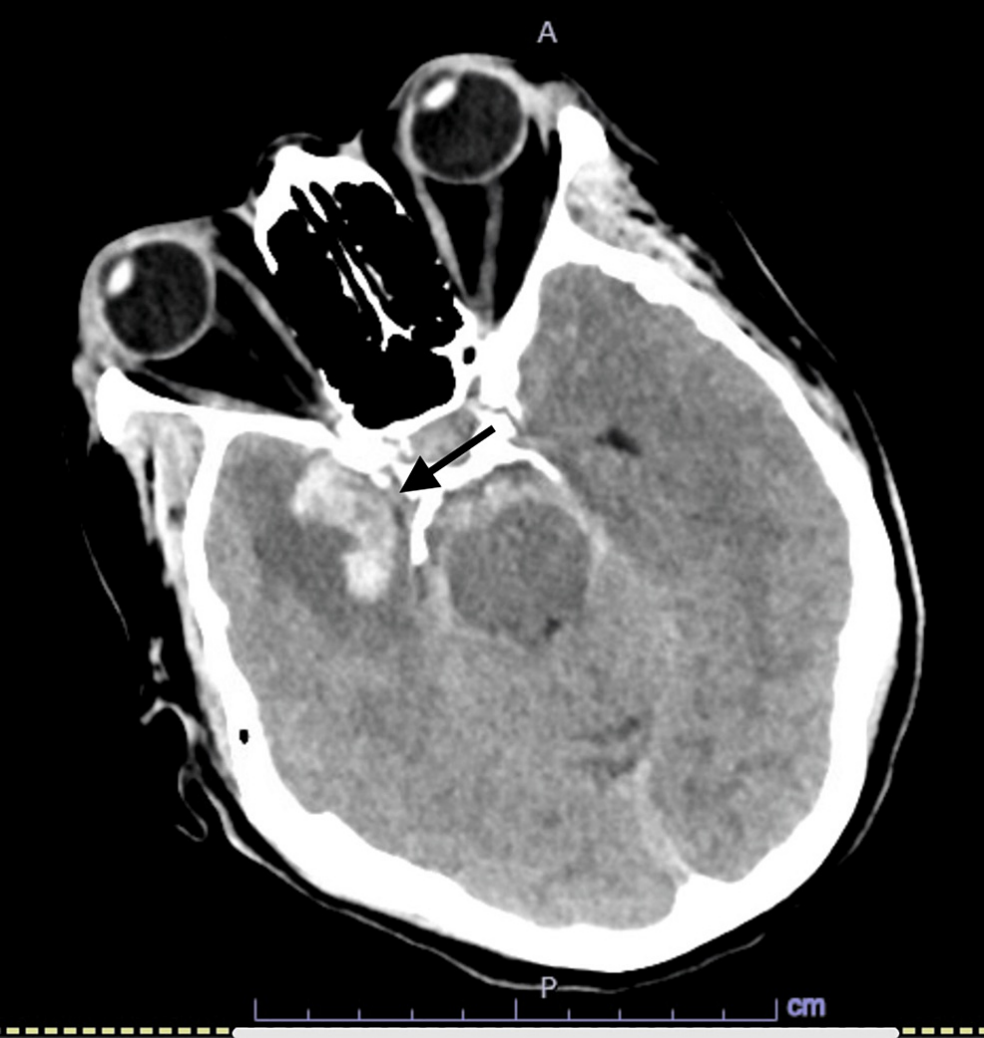
CT Head Computed tomography (CT) head showing evidence of right-sided subarachnoid hemorrhage (arrow).

**Figure 2:**
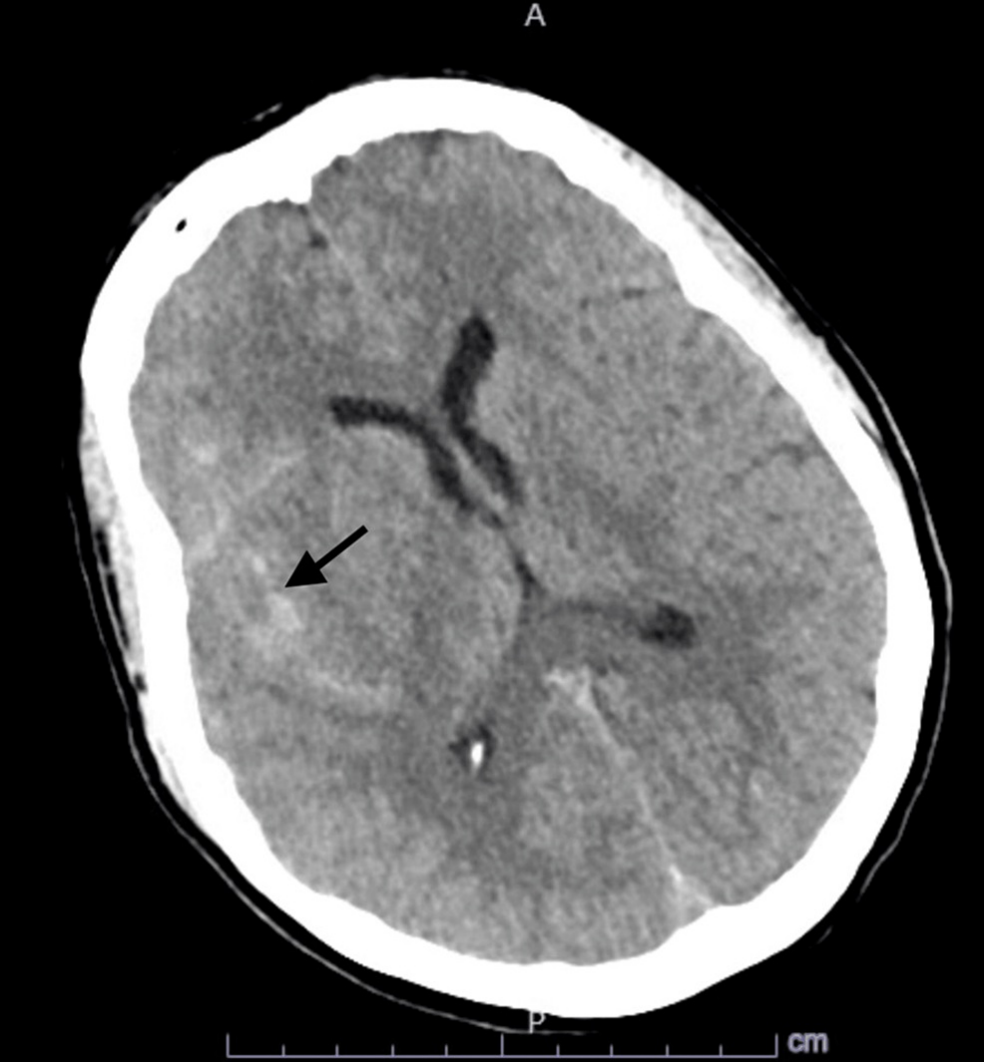
Parenchymal Extension of SAH Computed tomography (CT) head showing intraparenchymal extension of subarachnoid hemorrhage (SAH).

**Figure 3:**
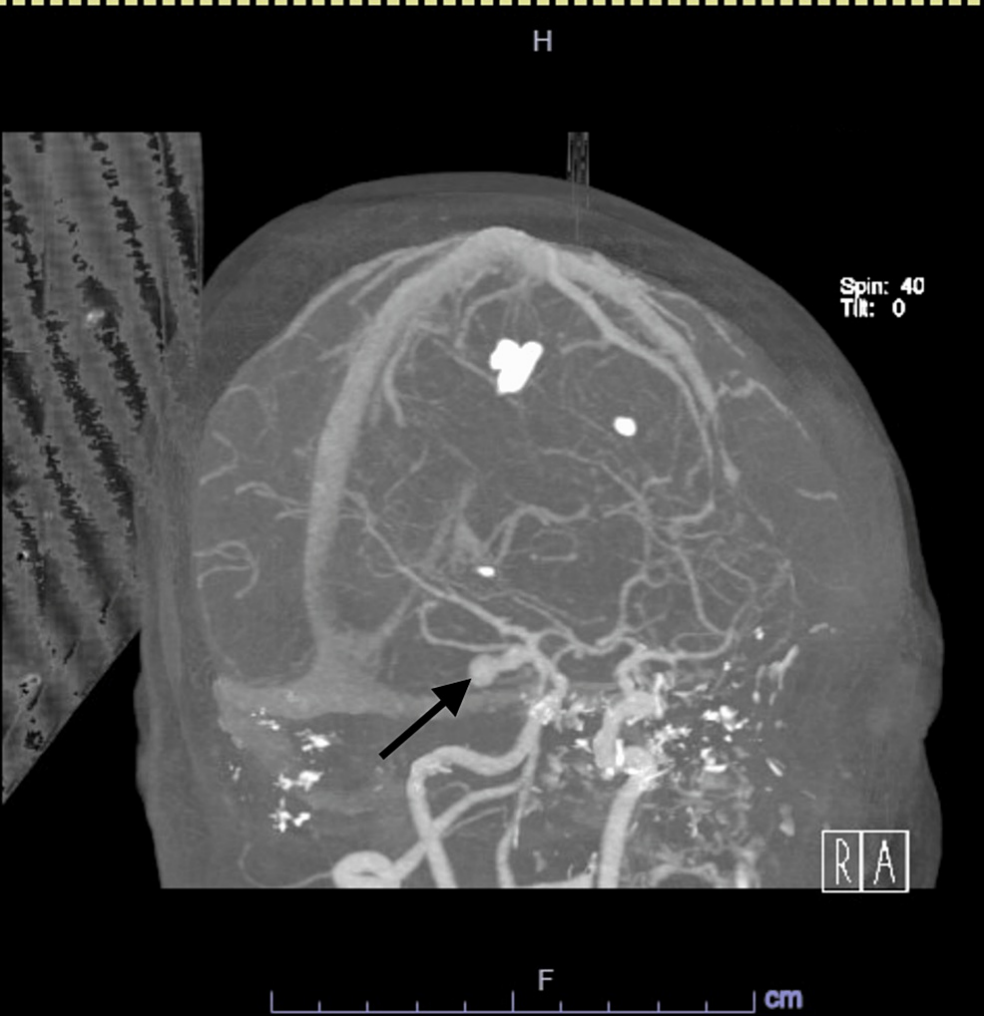
CTA Brain Computed tomography angiography (CTA) brain showing a multilobulated aneurysm formation affecting the right posterior communicating artery (arrow).

**Figure 4:**
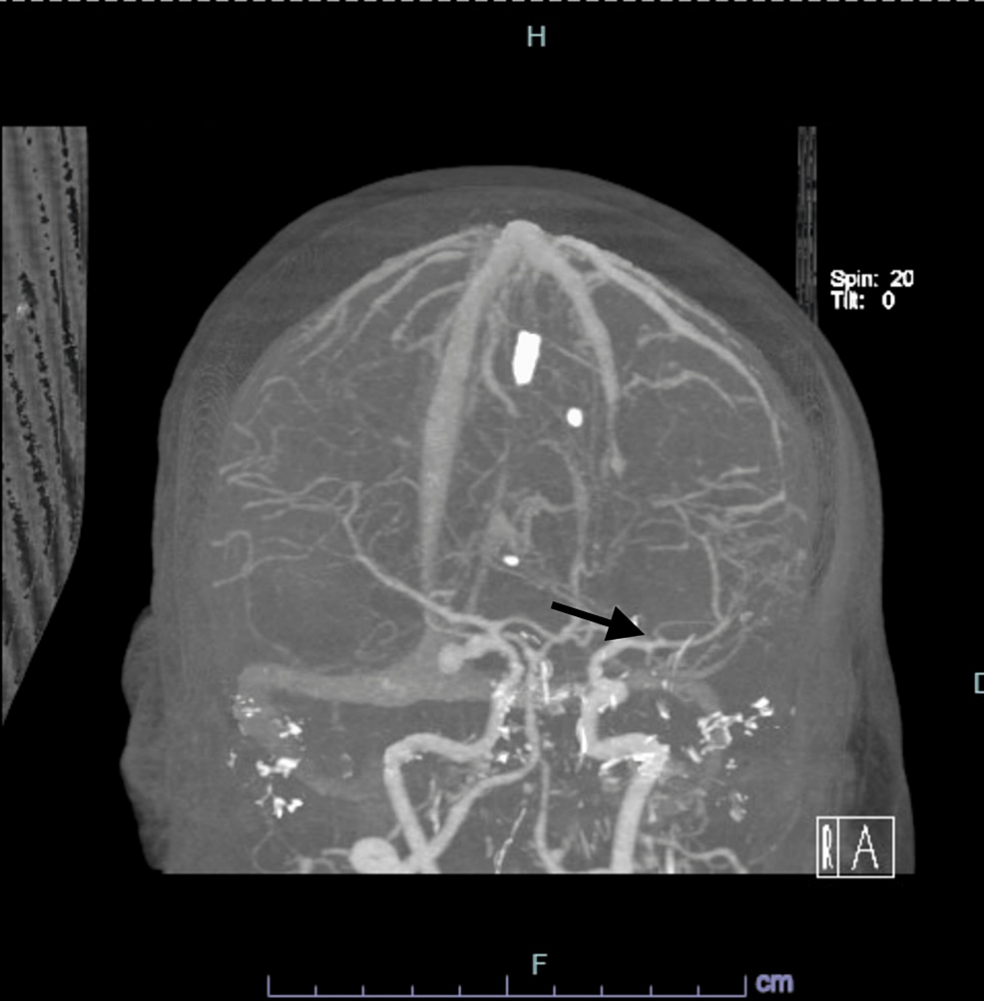
Left Posterior Communicating Artery Aneurysm Computed tomography angiography (CTA) brain showing evidence of a small aneurysm affecting the origin of the left posterior communicating artery (arrow).

A 61-year-old female patient presented to the emergency department (ED) with a severe headache associated with nausea and vomiting after a fall at home a few hours prior to the presentation. She also complained of left-sided upper and lower extremities weakness with involvement of the left facial side. Past medical history was significant for diabetes on metformin, primary SS with no active treatment, and CD in remission on adalimumab 80 mg every other week. In ED, vital signs included a temperature of 36.9 degrees Celsius, heart rate of 96 beats per minute, blood pressure of 105/55 mmHg, and SpO_2_ of 96% on room air. On physical examination, she was diaphoretic and in distress but alert, fully oriented, with no dysarthria, intact extraocular movement with no nystagmus, symmetrical facial movements, pupils equal and reactive bilaterally, muscle power of 5/5 on all extremities with intact deep tendon reflexes as well as sensation to light touch all over the body. Basic lab workup was remarkable only for leukocytosis at 12,000 cells/cubic ml. Computed tomography (CT) head showed SAH seen in the right Sylvian fissure, basal cisterns, and cerebral sulci with parenchymal extension with surrounding vasogenic edema (Figures 1-2). CT angiography (CTA) head showed a multilobulated right posterior communicating artery (PCOM) aneurysm along with a small aneurysm at the origin of the left PCOM (Figures 3-4). She also tested positive for COVID-19 infection and started on three days of remdesivir. However, her infection was mild, and she did not require supplemental oxygen throughout her hospitalization.

Diagnostic cerebral angiogram and coiling of the right PCOM aneurysm were pursued and she was started on nimodipine 60 mg every 4 hours. Successful coiling was achieved with complete obliteration of the dome and minimal residual neck. Angiogram also showed a 1 mm aneurysm of the right anterior choroidal artery. Continuous electroencephalography (EEG) monitoring showed no evidence of seizure activity and levetiracetam was discontinued. Further CT head showed minimal residual SAH, and she remained clinically stable and was discharged two weeks after coiling.

## Discussion

The incidence of CNS disorders in ulcerative colitis (UC) and CD is less than 0.5% of cases [2]. The incidence of all vascular complications in inflammatory bowel disease (IBD) is around 1.3% of cases [7]. These are seen less commonly in CD as compared to UC [8]. Like other extraintestinal manifestations of CD, cerebral manifestations are not reliably associated with the severity of intestinal disease [9]. Only a few case reports of cerebral aneurysms in association with CD have been described [10]. In addition, CD has been described in association with vascular aneurysms elsewhere e.g., the jejunal artery [11]. Although the underlying mechanism has been largely unknown, direct toxic neurological effects of inflammatory mediators or cerebral vasculitis have been suggested. Cerebral vasculitis has been histologically confirmed only in one case [12]. Segmental arterial mediolysis, characterized by lysis of tunica media resulting in media and adventitia separation with pseudoaneurysm formation, has also been reported in association with CD and SAH [13].

CNS involvement in SS has been reported in up to 68% of cases [6]. Manifestations can be diffuse like cognitive deficits, meningoencephalitis or focal resembling stroke, multiple sclerosis, or neuromyelitis optica [14]. The underlying mechanism of CNS involvement in SS is likely vasculitis, which can be associated with vascular occlusion and the development of collateral vessels known as quasi-moyamoya vessels that are prone to aneurysm formation and hemorrhage [15]. Several cases of SAH/cerebral aneurysms have been reported in association with SS [16,17].

Tumor necrosis factor (TNF) alpha is believed to have pro-inflammatory features that contribute to the growth and rupture of aneurysms [18]. Tumor necrosis factor alpha receptor 1 (TNFR1) has been recently suggested as a marker of the presence of aneurysms in those with acute SAH [19]. A retrospective study of 80 patients showed that TNFR1 has a sensitivity of 70.7% [19]. There is a theoretically beneficial effect of TNF alpha inhibitors including infliximab and adalimumab on the growth and progression of the aneurysm, although not demonstrated in cerebral vasculature [20]. These are presumed to affect the endothelial dysfunction and inflammatory process involved in aneurysm growth and expansion [20].

The association between COVID-19 infection and spontaneous SAH has also been suggested in the literature [1]. It was suggested that COVID-19 induces endothelial injury and local hypercoagulability secondary to the release of the Tissue Factor, leading ultimately to fibrinolytic pathway activation and increased risk of bleeding [21,22].

## Conclusions

Aneurysmal SAH should always be suspected in those with CD or SS who have sudden onset headaches and/or neurological deficits in the setting of COVID-19 infection. Both CD and SS have been associated with cerebral aneurysms. COVID-19 infection might act as a trigger of aneurysmal rupture and SAH. As was shown in this case, neurological manifestations do not correlate with the severity of intestinal CD or lacrimal/salivary SS.
